# An analysis of knowledge, attitudes, practice and influencing factors for tuberculosis prevention and control among Hainan University students

**DOI:** 10.3389/fpubh.2025.1478251

**Published:** 2025-01-15

**Authors:** Huifang Xie, Wencai Wang, Xuan Chen, Dan Huang, Qiuyue Yu, Liumei Luo

**Affiliations:** ^1^Hainan Vocational University of Science and Technology, Haikou, China; ^2^International Nursing School, Hainan Medical University, Haikou, China; ^3^TCM Department, Geriatric Hospital of Hainan, Haikou, China; ^4^Gynecology Department, Changzhi People’s Hospital, Changzhi, China; ^5^Department of Scientific Research, Hainan General Hospital, Haikou, China

**Keywords:** tuberculosis, KAP, students, prevention, influencing factors

## Abstract

**Objective:**

To assess the current status of knowledge, attitude, and practice pertaining to tuberculosis prevention among college students in Hainan Province, China, and to identify influential factors. The findings of this study are intended to provide valuable insights for the development and implementation of effective health education programs aimed at tuberculosis prevention and control.

**Methods:**

A convenient sampling method was employed to conduct a questionnaire-based survey among college students at a university in Hainan Province using the Wenjuanxing platform from January to February 2023. The survey evaluated students’ general information and their knowledge, attitude, and practice regarding tuberculosis prevention and control. The scores of correct knowledge, attitude, and practice were compared based on students’ demographic characteristics. Multiple linear regression analysis was used to determine the influencing factors of students’ knowledge, attitude, and practice related to tuberculosis prevention and control.

**Results:**

A total of 280 questionnaires were distributed, of which 265 valid responses were collected, resulting in a valid response rate of 94.6%. The study found that medical students exhibited higher levels of correct knowledge, attitude, and practice compared to non-medical students (5.92 vs. 5.17, 3.17 vs. 2.57, 3.48 vs. 2.78, respectively). Moreover, students who had received tuberculosis education had higher correct scores compared to those who had not (5.92 vs. 5.31, 3.08 vs. 2.65, 3.31 vs. 2.93, respectively). Additionally, students in higher grades demonstrated higher scores in attitude. Three variables were found to influence students’ knowledge, attitude, and practice related to tuberculosis prevention and control, accounting for 33.4% of the explanatory power (*F* = 11.930; *p* < 0.001). Grade, major, and whether they had received tuberculosis education included.

**Conclusion:**

The study revealed a generally poor level of awareness among college students regarding tuberculosis prevention and control knowledge and the importance of regular physical exercise. Grade, major, and whether students had received tuberculosis education were identified as influencing factors. These factors should be prioritized in university tuberculosis education programs. Moreover, the implementation of physical education in schools is crucial in promoting students’ health.

## 1 Background

Tuberculosis (TB) is a disease caused by the pathogenic bacterium *Mycobacterium tuberculosis*. It is commonly referred to as “active” TB or TB “disease” in order to distinguish it from TB infection (1). TB represents a significant global public health concern, posing a serious threat to human health. Particularly in Ethiopia and other sub-Saharan African countries, TB has emerged as the leading cause of mortality in adults, contributing to as much as 16.8% of deaths (2). According to the World Health Organization’s TB Report in 2021, there were 9.87 million new cases of pulmonary TB reported internationally in 2020, with China accounting for 8.5% of these cases (3).

In educational settings, the close proximity and frequent interactions among individuals create an environment conducive to the transmission of TB (4). The knowledge, attitude, and practice (KAP) of students with regard to TB prevention and control have been found to be closely linked to the incidence of TB (5). Particularly, adolescents, including university students, have emerged as a high-risk demographic for TB in China, largely due to their limited awareness of TB prevention and control measures (6). Notably, a national survey conducted in 2016 revealed that the awareness rate of essential TB prevention and control knowledge among college students was only 61.40% (7). Furthermore, a study conducted in Indonesia demonstrated that students majoring in health and who received specific education on TB exhibited greater knowledge, improved attitude, and more favorable practices towards TB prevention (4). This pattern was also observed in health students compared to their non-health counterparts across various regions such as Afghanistan and Jordan (8, 9). Moreover, students who received information on TB demonstrated enhanced behavioral responses to TB. Additionally, gender, academic grade, and place of origin have been identified as influential factors in shaping students’ KAP towards TB prevention and control (10–12). Research in Pakistan found female were better than male in knowledgeable about TB (10). Specifically, research conducted in Pakistan reported that females exhibited higher levels of knowledge about TB compared to males. Similarly, studies conducted in China revealed that third-year students exhibited higher knowledge and practice scores towards tuberculosis in comparison to first- and second-year students (11). Additionally, a cross-sectional study in Tanzania found that rural participants displayed greater knowledge, more positive attitudes, and better practices towards TB compared to their urban counterparts (12). Moreover, in southern Thailand, a cross-sectional study found that the educational status of parents exerted an impact on students’ KAP related to dengue fever (13).

As a result of its proximity to the equator, Hainan Province experiences a distinct tropical climate characterized by elevated temperatures and heightened water vapor pressure, factors which contribute to an increased risk of TB transmission (14). Consequently, TB holds the highest incidence ranking among class A and B infectious diseases in Hainan Province, with the mortality rate following closely in second position (15). Given the interplay of Hainan’s tropical climate and the university’s notable population density, TB prevention and control have emerged as prominent public health concerns within the region. To address this, the present study endeavors to gain insights into the KAP pertaining to TB prevention among college students in Hainan Province through the administration of a questionnaire survey. The findings of this research are aimed at providing valuable insights to inform the development of targeted health education initiatives focused on tuberculosis prevention and control.

## 2 Methods

### 2.1 Study design and participants

A convenience sampling method was applied to carry out a questionnaire survey targeting college students at a university in Hainan Province from January to February 2023. At the first, researchers designed the electronic questionnaire and published through the Wenjuanxing platform. Secondly, researchers conducted on-site interception visits to students in the school canteen, activity center, dormitory building and other places. Then, the students be questioned according to the inclusion and exclusion criteria, and identified the students meet the inclusion criteria. At the last, researchers issued electronic questionnaires to that students to fill out.

WenJuanxing is a specialized online questionnaire survey platform that offers users humanized online data collection, survey results analysis, and other related services. It boasts the advantages of rapid data collection and low cost, and is extensively utilized in academic institutions, businesses, and by individuals.

The inclusion criteria were as follows: (1) individuals aged 16 years or above; (2) enrolled as full-time students at the university; (3) registered students of Hainan University of Science and Technology; and (4) willingness to volunteer for the study. Conversely, the exclusion criteria encompassed: (1) students who are suspended or have discontinued their studies; and (2) individuals unable to complete the survey within the stipulated timeframe.

### 2.2 Determination of sample size

When exploring the influencing factors of related variables, the minimum sample size is typically recommended to be 5–10 times the number of variables in the study (16). Given that the questionnaire used in this study contains 25 items, accounting for a 10% margin for potential invalid responses, a planned distribution of 25*10*(1 + 10%) = 275 questionnaires is proposed.

### 2.3 Survey tool

The questionnaire was meticulously crafted by the researchers, drawing on extensive literature review and consultations with experts in the field, including school physicians, epidemiologists, and CDC staff. Structurally, the questionnaire comprises three parts, focusing on participants’ basic information (8 questions), information pertaining to TB prevention and control (8 questions), and attitudes (4 questions) and practices (5 questions) related to TB prevention and control. To test the questionnaire, a pilot study was conducted in Dec 2022 among first, second, and third year medical and non-medical students. The questionnaire’s internal consistency was assessed using Cronbach’s *α* coefficient, yielding a value of 0.960, indicating high reliability. Additionally, the Kaiser-Meyer-Olkin (KMO) test, which produced a KMO value of 0.814, confirmed the questionnaire’s satisfactory reliability and validity.

#### 2.3.1 Characteristics of participants

The first section of the questionnaire pertains to the demographic information of the survey participants, encompassing gender, academic year, birthplace, field of study, exposure to TB prevention and control education, as well as parental education level. Gender and birthplace categories were determined in accordance with the information provided in the participants’ household registration documents, with gender classified as male or female and birthplace classified as either rural or urban. Academic year and field of study classifications were derived from the students’ academic records. Most fields of study are four-year programs, with select disciplines, such as nursing, extending to five years. As such, academic years are categorized as freshman, sophomore, junior, and senior or higher, while fields of study are classified as either medical or non-medical. Exposure to TB prevention and control education was determined based on whether participants had received training from educational or medical institutions, including schools, hospitals, centers for disease control, or other relevant healthcare and educational entities. Finally, parental education level was assessed using the national education code standard (GB4658-84), and categorized as junior high school or below, high school/vocational school/junior college, and undergraduate level and above, which serves as an indicator of their cultural attainment.

#### 2.3.2 TB prevention and control information

The second section of the questionnaire pertains to TB prevention and control information, drawing upon the Core Information on TB Prevention and Control Knowledge issued by the Chinese Center for Disease Control and Prevention in 2016 (7). With the aim of disseminating knowledge on TB prevention and control, the National Health and Family Planning Commission convened experts to deliberate and compile the core information. This section comprises 8 items, which assess the awareness of core knowledge of TB prevention and control among college students.

#### 2.3.3 Attitudes and practices regarding TB prevention and control

The third section of the questionnaire concerns attitudes and practices regarding TB prevention and control, and draws upon the research conducted by Jiang et al. (17), comprising nine items. The Cronbach’s *α* coefficient, split-half reliability coefficient, and test–retest reliability coefficient were calculated to be 0.86, 0.78, and 0.91, respectively. This section delves into the attitudes and practices of college students in relation to TB prevention and control, with four items dedicated to attitudes and five items pertaining to practices in this context.

For the questions, participants were required to answer “Yes” or “No”. Each correct answer had a score of 1, while the wrong answer had a score of 0.

### 2.4 Calculation formula

Single Item Knowledge Awareness Rate = Number of individuals who answered the item correctly/Total number of survey respondents ×100%; Correct Knowledge Awareness Rate = Number of correctly answered questions/Total number of questions×100%. According to the given criteria by Chinese CDC, the benchmark for compliance is: Correct Knowledge Awareness Rate > 80%.

### 2.5 Statistical analysis

The data collected through the electronic platform is the original data, and the results are presented as item options that need to be transformed for statistical analysis. Data entry was carried out by two skilled individuals utilizing EpiData 3.1 software, whereas the subsequent analysis of the data was performed using SPSS 26.0 software.

Descriptive statistics such as frequency and percentage were employed to delineate count data. Separate scores were calculated for questions related to knowledge, attitude, and behaviors. Score mean (SM) and standard deviation (SD) were computed for different subgroups on the basis of various categories. In order to analyze the disparities in the knowledge, practice, and attitudes concerning TB prevention and control amongst students from various categories, T-test and ANOVA analysis was undertaken. Statistical significance was determined at a significance level of *p* < 0.05.

Using the number of correctly answered items as the dependent variable and participants’ basic information as the independent variables, a multiple linear regression model was employed to examine the influencing factors of KAP relating to tuberculosis prevention and control among students. Gender (1 = male, 2 = female), grade (1 = freshmen, 2 = sophomores, 3 = juniors, 4 = seniors and above), place of origin (1 = rural areas, 2 = town areas), major (1 = medical, 2 = non-medical), whether received TB education (1 = yes, 2 = no), father/mother’s educational level (1 = junior high school and below, 2 = high school/vocational school/junior college, 3 = Undergraduate and above) are assigned as categorical variable and incorporated into the model as a dummy variable. A two-sided test was conducted at a significance level *α* = 0.05 to evaluate the model’s statistical significance.

### 2.6 Ethical considerations

The studies involving human participants were reviewed and approved by the Medical Ethics Committee of Hainan General Hospital (no. Med-Eth-Re [2022]752). Written informed consent to participate in this study was provided by the participants and, for participants under the age of 18, written informed consent to participate in this study was provided by the participants’ legal guardian/next of kin.

## 3 Results

### 3.1 Basic information

In this study, a total of 280 questionnaires were distributed to participants, yielding 265 valid responses, indicating an effective response rate of 94.6%. The sample comprised 136 male and 129 female respondents. The respondents were all over 18 years old, with an average age of (21.58 ± 0.96) years. Geographically, 107 respondents were from rural areas, and 158 were from urban areas. Additionally, there were 121 respondents majoring in medical fields and 144 in non-medical fields. Detailed demographic information is presented in Table 1.

**Table 1:** Comparison of TB-related knowledge awareness rates among different demographic groups.

Items		Number	Percentage(%)
Gender	Male	136	51.32
	Female	129	48.68
Grade	Freshmen	56	21.13
	Sophomores	46	17.36
	Juniors	65	24.53
	Seniors and above	98	36.98
Place of origin	Rural areas	107	40.38
	Town areas	158	59.62
Major	Medical	121	45.66
	Non-medical	144	54.34
Received TB education	Yes	119	44.90
	No	146	55.10
Father’s educational level	Junior high school and below	74	27.92
	High school/Vocational school/Junior college	103	38.87
	Undergraduate and above	88	33.21
Mother’s educational level	Junior high school and below	82	30.94
	High school / Vocational school / Junior college	96	36.23
	Undergraduate and above	87	32.83

### 3.2 Response to core knowledge of TB prevention and control

The findings reveal that the accurate knowledge awareness rate of college students regarding TB prevention and control is 46.79%. Of the 8 items assessing core knowledge of TB prevention and control among college students, the top three items with the highest correctness rates were “Older adult people and individuals with low immunity are susceptible to TB,” “The main mode of transmission of TB is through respiratory spread,” and “Individuals with symptoms suspicious of TB, such as coughing, sputum production for more than 2 weeks, or coughing up blood, should promptly seek medical attention at TB prevention and control institutions,” with correctness rates of 90.48, 85.71, and 80.95%, respectively. Conversely, the item with the lowest correctness rate was “The main strategy for TB prevention is to detect and treat infectious patients,” with a correctness rate of 36.81%. Detailed findings are presented in Table 2.

**Table 2:** Correctness of core TB prevention and control knowledge among college students.

Items	Correct number	Correct rate(%)
Older adult individuals and those with weakened immune systems are susceptible to TB	239	90.48
The main mode of transmission of TB is through respiratory droplets	225	85.71
Individuals who have had symptoms suspicious of TB, such as coughing, producing sputum for more than 2 weeks, or coughing up blood, should promptly seek medical attention at TB prevention and control institutions	212	80.95
TB is caused by Mycobacterium TB	201	76.19
Tuberculosis is seriously harmful to health for a long time	193	73.16
In the “Law of the People’s Republic of China on the Prevention and Control of Infectious Diseases,” TB is classified as a Class B infectious disease	174	66.61
The principles that must be followed in anti-TB treatment are early, combined, appropriate dosage, regularity, and full course	140	53.19
The primary strategy for TB prevention is to detect and treat infectious patients	95	36.81

### 3.3 Attitudes and practices regarding TB prevention and control

The findings from the survey of college students revealed that, out of the 9 items related to attitudes and practices in TB prevention and control, the three items with the highest correctness rates were as follows: “Students and teachers should proactively report suspected symptoms or diagnosis of TB and avoid concealing them,” “Avoid indiscriminate spitting,” and “Cover the mouth and nose with hands or a tissue when coughing or sneezing,” with correctness rates of 85.66, 84.90, and 79.62%, respectively. In contrast, the item with the lowest correctness rate was “Regular physical exercise,” with a correctness rate of 36.98%. Further details can be found in Table 3.

**Table 3:** Attitudes and behaviors of college students regarding TB prevention and control.

Items	Correct number	Correct rate (%)
Attitude		
College students need to acquire knowledge about TB prevention and control	155	58.49
Schools should conduct health education on TB prevention and control	173	65.28
Teachers and students who have suspected symptoms of TB or have been diagnosed with TB should actively report to their teachers and not conceal the information	227	85.66
Teachers and students should not reject individuals with TB	198	74.72
Behavior		
The dormitories should be ventilated by opening windows for at least half an hour every day	173	65.28
Do not spit indiscriminately	225	84.90
Regular physical exercise	98	36.98
Cover mouth/nose with hands/tissue when coughing or sneezing	211	79.62
Proactively learn about TB prevention knowledge	115	43.40

### 3.4 Comparison of correct KAP regarding tuberculosis prevention and control among college students with diverse characteristics

The findings reveal that students majoring in medical disciplines and those who have received tuberculosis health education exhibit higher levels of adherence to correct KAP concerning TB prevention and control. Furthermore, as college students progress through their academic years, they show increasing compliance with standards for correct attitudes towards TB. Additionally, students with parents possessing higher levels of education display greater adherence to standards for correct practice. These disparities are statistically significant (*p* < 0.05). However, there were no statistically significant differences observed in the levels of adherence to correct KAP among college students of different genders and ethnic backgrounds (*p* > 0.05). Refer to Table 4 for further details.

**Table 4:** Score mean (SM) and standard deviation (SD) of students’ TB knowledge, attitude, and behavior based on different characteristics.

Different characteristics	Statistical values	Knowledge	Attitude	Behavior
Gender				
Male	SM(SD)	5.52(1.65)	2.89(1.34)	2.93(1.51)
Female		5.64(1.71)	2.79(1.55)	3.28(1.60)
	t	−0.587	0.558	−1.809
	*P*	0.558	0.578	0.072
Grade				
Freshmen	SM(SD)	5.36(1.80)	2.63(1.57)	2.88(1.49)
Sophomores		5.24(1.96)	2.22(1.55)	2.96(1.48)
Juniors		5.74(1.61)	3.09(1.31)	3.32(1.52)
Seniors and above		5.77(1.48)	3.09(1.31)	3.15(1.66)
	F	1.561	5.163	0.999
	*P*	0.199	0.002*	0.394
Place of origin				
Rural areas	SM(SD)	5.78(1.59)	2.79(1.47)	3.28(1.50)
Town areas		5.45(1.73)	2.88(1.43)	2.98(1.59)
	t	1.556	−0.524	1.537
	*P*	0.121	0.601	0.126
Major				
medical	SM(SD)	5.92(1.32)	3.17(1.29)	3.48(1.54)
Non-medical		5.17(1.95)	2.57(1.51)	2.78(1.51)
	t	−3.708	3.416	3.697
	*P*	0.000*	0.001*	0.000*
Whether Received TB education				
YES	SM(SD)	5.92(1.59)	3.08(1.39)	3.31(1.71)
No		5.31(1.71)	2.65(1.46)	2.93(1.41)
	t	2.974	2.407	1.981
	*P*	0.003*	0.017*	0.049*
Father’s educational level				
Junior high school and below	SM(SD)	5.51(1.68)	2.62(1.46)	2.76(1.55)
High school/Vocational school/Junior college		5.72(1.67)	2.77(1.53)	2.73(1.50)
Undergraduate and above		5.48(1.70)	3.11(1.29)	3.83(1.39)
	F	0.571	2.594	15.972
	*P*	0.566	0.077	0.000*
Mother’s educational level				
Junior high school and below	SM(SD)	5.59(1.71)	2.78(1.42)	2.95(1.56)
High school/Vocational school/Junior college		5.67(1.65)	2.68(1.57)	2.57(1.49)
Undergraduate and above		5.48(1.70)	3.08(1.30)	3.83(1.37)
	F	0.273	1.904	17.200
	*P*	0.762	0.151	0.000*

### 3.5 Result of multiple linear regression model

Table 5 displays the outcomes of a linear multiple regression analysis, which identifies the variables that impact students’ KAP regarding tuberculosis prevention and control. The analysis revealed that three variables significantly influenced students’ KAP in this area, accounting for 33.4% of the variance (*F* = 11.930; *p* < 0.001). These influential variables were Grade (*β* = 0.794), major (β = 0.545), and whether the students had received tuberculosis education (β = 1.260). Specifically, students in higher grades, those majoring in health-related fields, and those who had received TB education demonstrated better KAP in TB prevention and control.

**Table 5:** Variables affecting students’ KAP of TB prevent and control.

Variables	B^a^	SE	β	t	*P*
Constant	10.724	1.721	-	6.230	0.000*
Gender	0.503	0.481	0.092	1.045	0.298
Grade	0.708	0.206	0.653	3.439	0.001*
Place of origin	0.580	0.501	0.103	1.158	0.249
Major	1.083	0.482	0.973	2.246	0.025*
Whether Received TB education	1.479	0.511	1.260	2.894	0.040*
Father’s educational level	0.283	0.757	0.083	0.373	0.710
Mother’s educational level	0.150	0.810	0.042	0.185	0.854
R^2^	0.331				
Adjusted R^2^	0.328				
F	*F* = 10.331, *p* = 0.000				

*Significant; ^a^ Unstandardized coefficient.

## 4 Discussion

### 4.1 Analysis of college students’ knowledge status on TB prevention

The results of the study indicate that the accurate knowledge awareness rate of college students regarding TB prevention and control is only 46.79%, significantly lower than the target value of 80% set by the National Health Commission (18), which is consistent with the findings of Wu T and others (19, 20). Given that college students are among the high-risk groups for TB, the densely populated nature of college campuses poses potential serious consequences in the event of TB outbreaks (21, 22). Additionally, the unique tropical climate of Hainan Province, characterized by high temperatures and high water vapor pressure, can elevate the risk of TB incidence and transmission (14). Educating college students in Hainan Province about TB prevention and control is therefore paramount for establishing a robust campus health and safety system and enhancing public health safety management. Public health prevention agencies and campus administrators should prioritize the education of college students on TB prevention and control and implement diverse and efficient educational activities on campus. In light of the survey results, targeted promotional and educational initiatives should be developed, and regular educational activities should be organized for college students on campus, such as knowledge quizzes with incentives and community service engagement.

### 4.2 Analysis of students’ attitudes and practice status toward TB prevention

The study revealed that there is room for improvement in the overall habitual exercise patterns of college students. The research findings indicate that the aspect with the lowest adherence rate among college students in relation to TB prevention and control is “regular physical exercise,” standing at a mere 36.98%. This implies that the overall practice of regular exercise among college students is subpar. Telford DM contends that inadequate physical activity and prolonged sedentary practice have emerged as significant factors compromising the health of young individuals (22, 23). The “Chinese Children and Adolescents Physical Activity Guidelines” recommend a minimum of 60 min of moderate to vigorous physical activity daily to enhance the physical well-being of young people (24). The “Outline of Building a Strong Sports Nation” advocates for prioritizing school-based physical education as a key strategy for promoting youth health and implementing plans to bolster sports activities among the youth (25). Therefore, educational institutions should advocate the principle of “enhancing physical fitness through sports and building a robust nation through sports,” and fortify the physical well-being of young individuals through exercise to strengthen the campus health management system. College students are confronted with academic, employment, and emotional stressors, which contribute to their lackluster daily exercise habits (26). Hence, society and educational institutions can integrate physical activity with emotional regulation and stress alleviation to motivate students and optimize the efficacy of exercise.

### 4.3 Single factor analysis of student characteristics and their KAP

#### 4.3.1 Educational intervention

The findings of this study indicate that students majoring in medicine and those who have received education on tuberculosis (TB) exhibit higher levels of compliance with accurate KAP related to TB prevention and control, with statistically significant differences (*p* < 0.05). Interestingly, no significant discrepancies in compliance rates based on gender or place of origin were observed (*p* > 0.05), suggesting the need for TB prevention and control interventions to be inclusive of all students, irrespective of demographic factors. The Knowledge-Attitude-Practice (KAP) theory asserts that the accumulation of knowledge leads to the formation of attitude, subsequently influencing practice. Knowledge serves as the foundation, attitude as the impetus, and practice as the ultimate objective (27, 28). When compared to their peers, students majoring in medicine and those who have received TB health education have been exposed to training programs centered on TB prevention and control, resulting in a more comprehensive understanding of accurate TB prevention and control knowledge, as well as more scientifically informed attitudes and practices pertaining to TB prevention and control (4, 29). Consequently, through systematic and science-based knowledge training, incorporating health education, lectures, and classroom instruction, college students can be guided to develop accurate beliefs regarding TB prevention and control, thereby fostering relevant health practices. Furthermore, as significant conveyors of ideas and influencers of societal practice, college students’ KAP concerning disease prevention and control exert a direct impact on public understanding, beliefs, and practices relating to diseases, indirectly influencing the formulation and adaptation of disease prevention and control policies by health management authorities. Therefore, the training of college students can be instrumental in promoting the execution and enhancement of disease prevention and control strategies.

#### 4.3.2 The influence of time on practice development

The findings of the research indicate a positive correlation between college students’ academic performance and their adherence to accurate attitudes towards TB prevention and control. It was also observed that higher parental education levels corresponded to increased rates of compliance with correct practices, with statistically significant variances (*p* < 0.05). Over time, the entrenched influence of the family environment may have shaped college students’ practices in ways that did not promote healthy lifestyle habits, such as neglecting to cover their mouth or nose when coughing or sneezing, or adopting detrimental practices like public spitting. Upon entering college, these pre-existing habits may prove resistant to immediate change. Conversely, the dissemination of health-related information within the campus setting challenges these entrenched beliefs, and on-going health education endeavors to reframe them, ultimately leading to the establishment of healthier practices. Human behavior development theory posits that the cultivation of healthy behaviors is greatly influenced by the presence of healthy lifestyle habits (30). These habits are characterized as patterned reactions exhibited in specific contexts over an extended period (31). Given that the family sphere serves as the prolonged milieu for adolescent maturation, it wields considerable influence in shaping habitual behaviors. Moreover, parental conduct pertaining to TB prevention and control subtly impacts the behavioral patterns of college students with regard to the same (32, 33). Simultaneously, college students find themselves in a transitional phase as they begin to assert independence from their parental influences, while also being subject to the educational guidance delivered by academic institutions, which serves to adjust, modify, and reshape pre-existing beliefs (6, 34). Thus, the inculcation of healthy behaviors in adolescents requires thoughtful examination of the combined impact of familial background and scholastic environment, as well as an astute appreciation for the role of time in the process of translating beliefs into behavior. The establishment of healthy habits demands the consistent performance of healthy behaviors over an extended duration.

### 4.4 The multiple linear regression analysis of student characteristics and their KAP towards tuberculosis prevention and control

The findings of the multiple linear regression analysis revealed significant associations between Grade (*β* = 0.794), major (β = 0.545), and the reception of tuberculosis (TB) education (β = 1.260) with student KAP scores in relation to TB prevention and control. Specifically, the results indicate that students in higher grade levels, those majoring in health-related fields, and those who have received TB education exhibit better KAP towards TB prevention and control. This aligns with the outcomes of previous research. Notably, a study conducted at a Jordanian university demonstrated that students enrolled in healthcare disciplines exhibited significantly higher KAP scores (9). This can be attributed to the inclusion of infectious disease education, including TB, in the healthcare curriculum, thereby enhancing students’ preparedness in tuberculosis prevention (14). Furthermore, healthcare students face an increased risk of TB exposure during their work in healthcare settings, which motivates them to actively seek out knowledge related to TB prevention (34). Consequently, students who have received TB education are found to possess superior KAP compared to those who have not. This finding is corroborated by the work of researcher Hassan AO, who similarly concluded that TB education positively influences students’ KAP related to tuberculosis (35). Based on these insights, it is recommended that universities devise interventions aimed at enhancing students’ KAP of TB prevention, potentially through integrating disease education with their course materials. Additionally, it was observed that students at higher academic levels exhibited better KAP than those at lower levels, as supported by a cross-sectional study in Namibia, which identified the student’s year of study as a significant factor influencing TB knowledge (36). Similarly, a survey conducted at Taif University found that students in the earlier stages of their academic journeys had inadequate KAP towards TB (22).

### 4.5 Deficiencies and prospects

This study has illuminated the KAP surrounding TB prevention and control among college students in Hainan Province. However, several limitations should be noted. Firstly, the sample size of 265 respondents may not provide sufficient grounds for generalizing the findings to the entire student population. In addition, the demographic composition of the sample may not accurately reflect the diverse population of the province. Furthermore, the reliance on self-reported data introduces the potential for response bias and lacks validation measures. The cross-sectional design of the study allows for a snapshot of TB-related perceptions but hinders the ability to assess changes over time or establish causal relationships. While the study offers valuable insights, it is imperative to address these limitations by utilizing larger, more diverse samples and longitudinal designs to enhance the reliability and relevance of future research in this area.

## 5 Conclusion

In Hainan Province, college students exhibit a limited level of awareness concerning TB prevention and control. To address this issue, a range of pedagogical approaches can be employed, including the delivery of health lectures, classroom education, and the dissemination of information online, with the aim of enhancing student KAP toward TB. Furthermore, given that educational institutions serve as primary settings for students’ daily activities and intellectual growth, it is imperative for schools to recognize the enduring influence of the environment on students’ KAP related to TB prevention. Consequently, schools should design tailored educational initiatives in accordance with the unique characteristics of their student body. For instance, universities might consider implementing fundamental TB knowledge training programs for lower-grade students, particularly those pursuing non-medical majors and who have not previously received instruction on TB.

## Data Availability

The datasets presented in this article are not readily available because they require all the authors’ agreement. Requests to access the datasets should be directed to chapeal@163.com.
