# Supplementary material for: An analysis of knowledge, attitudes, practice and influencing factors for tuberculosis prevention and control among Hainan University students
Source: Front Public Health. 2025 Jan 15;13:1478251. doi: 10.3389/fpubh.2025.1478251 (PMC11775896; doi:10.3389/fpubh.2025.1478251)
Supplement: Supplementary file 1 [file Table_1.docx]

Questionnaire on knowledge, attitude and behavior of tuberculosis prevention and control among college students in Hainan Province

Dear students,

Hello! Thank you for taking the time to participate in this survey. We are conducting a study on the status quo of knowledge, attitude and behavior of tuberculosis prevention and control among college students in Hainan Province, so as to provide reference for health education on tuberculosis prevention and control. This questionnaire consists of four parts: general information, core knowledge of tuberculosis prevention and control, attitude and behavior of tuberculosis prevention and control. All questions are single choice. We are committed to making this survey efficient and not taking up too much of your time, and we expect it to take approximately 10-15 minutes to complete.

We promise to keep your answers strictly confidential, and all data collected will be used only for statistical analysis and will not disclose your personal information. You can share your true thoughts with confidence. Your answer is very important to us, thank you for your support for this study!

**ⅠBasic information**

1. Age: years old
2. Gender

①Male

②Female

1. Grade

①Freshmen

②Sophomores

③Juniors

④Seniors and above

1. Place of origin

①Rural areas

②Town areas

1. Major

①medical

②Non-medical

1. Whether Received TB education

①YES

②No

1. Father's educational level

①Junior high school and below

②High school / Vocational school / Junior college

③Undergraduate and above

1. Mother's educational level

①Junior high school and below

②High school / Vocational school / Junior college

③Undergraduate and above

**Ⅱ TB prevention and control knowledge**

1. Elderly individuals and those with weakened immune systems are susceptible to TB

①YES

②NO

1. The main mode of transmission of TB is through respiratory droplets

①YES

②NO

1. Individuals who have had symptoms suspicious of TB, such as coughing, producing sputum for more than 2 weeks, or coughing up blood, should promptly seek medical attention at TB prevention and control institutions

①YES

②NO

1. TB is caused by Mycobacterium TB

①YES

②NO

1. Tuberculosis is seriously harmful to health for a long time

①YES

②NO

1. In the "Law of the People's Republic of China on the Prevention and Control of Infectious Diseases," TB is classified as a Class B infectious disease

①YES

②NO

1. The principles that must be followed in anti-TB treatment are early, combined, appropriate dosage, regularity, and full course

①YES

②NO

1. The primary strategy for TB prevention is to detect and treat infectious patients

①YES

②NO

**Ⅲ TB prevention and control Attitude**

1. College students need to acquire knowledge about TB prevention and control

①YES

②NO

1. Schools should conduct health education on TB prevention and control

①YES

②NO

1. Teachers and students who have suspected symptoms of TB or have been diagnosed with TB should actively report to their teachers and not conceal the information

①YES

②NO

1. Teachers and students should not reject individuals with TB

①YES

②NO

**Ⅳ TB prevention and control Practice**

1. The dormitories should be ventilated by opening windows for at least half an hour every day

①YES

②NO

1. Do not spit indiscriminately

①YES

②NO

1. Regular physical exercise

①YES

②NO

1. Cover mouth/nose with hands/tissue when coughing or sneezing

①YES

②NO

1. Proactively learn about TB prevention knowledge

①YES

②NO

The questionnaire is end. Thank you for your participation！
